# Increasing the effectiveness of the Diabetes Prevention Program through if-then plans: study protocol for the randomized controlled trial of the McGill CHIP Healthy Weight Program

**DOI:** 10.1186/1471-2458-14-470

**Published:** 2014-05-18

**Authors:** Bärbel Knäuper, Elena Ivanova, Zhen Xu, Melodie Chamandy, Ilka Lowensteyn, Lawrence Joseph, Aleksandra Luszczynska, Steven Grover

**Affiliations:** 1 Department of Psychology, McGill University, 1205 Dr. Penfield Avenue, Montreal H3A 1B1, QC, Canada; 2 Comprehensive Health Improvement Program, McGill University, 5400 Westbury Avenue, Montreal H3W 2W8, QC, Canada; 3 Department of Epidemiology and Biostatistics, McGill University, 687 Pine Avenue West, V Building, Montreal H3A 1A1, QC, Canada; 4 Department of Psychology, Trauma, Health, and Hazards Center of the University of Colorado, 1420 Austin Bluffs Parkway, University Office Center, Colorado Springs, CO 80933-7150, USA; 5 Department of Psychology, University of Social Sciences and Humanities, 30 Ostrowskiego St, 53238 Wroclaw, Poland

**Keywords:** Lifestyle modification program, Implementation intentions, Mental imagery, Mental practice, Eating behavior, Physical activity, Weight-loss, Chronic disease, Habit formation

## Abstract

**Background:**

The Diabetes Prevention Program (DPP) is highly effective in promoting weight loss in overweight and obese individuals. However, one-on-one DPP sessions are costly. As a cost-saving alternative, a group version of the DPP, called Group Lifestyle Balance program (GLB), has been developed but has been shown to be less effective. The aim of this two-arm parallel randomized controlled trial is to increase the effectiveness of the GLB by integrating habit formation techniques, namely if-then plans and their mental practice, into the program.

**Methods/Design:**

A total of 154 participants will be randomized to a standard or enriched GLB program. For the enriched GLB program, if-then plans and their mental practice will be integrated into the standard GLB program. Participants will be overweight or obese men and women (BMI of 28 to 45 kg/m^2^, waist circumference ≥ 88 for women, ≥ 102 for men, 18 to 75 years of age) who do less than 200 minutes of self-reported moderate or vigorous exercise per week. Measures will be completed at baseline, 3 months, post-intervention (12 months), and 12 months post-intervention (24 months). The primary outcome measure is weight loss at 3, 12, and 24 months. Secondary outcomes include percent reaching weight loss goal, physical activity at 3, 12, and 24 months, and weight-related risk factors (waist circumference, hemoglobin A1c, systolic/diastolic blood pressure, total cholesterol/HDL ratio). Standardized training of the life-style coaches, use of standardized manuals, and audio taping and reviewing of the sessions will ensure intervention fidelity.

**Discussion:**

The study will provide evidence-based data on the effectiveness of an enhanced GLB intervention in promoting weight loss and in reducing weight-related risk factors for chronic health problems. Ethical clearance has been received from the Research Ethics and Compliance Board of the Faculty of Medicine Research and Graduate Studies Office at McGill University (Montreal, Canada).

**Trial registration:**

ClinicalTrials.gov Identifier: NCT02008435. Registered 6 December 2013.

## Background

Being overweight (i.e., body mass index [BMI] ≥ 25 kg/m^2^) or obese (i.e., BMI ≥ 30 kg/m^2^) is one of the leading preventable causes of death in North America, with approximately 500,000 premature deaths each year in the US [1,2]. Overweight and obesity carry the risk of causing health complications, including type 2 diabetes, cardiovascular disease (e.g., heart attack, stroke), hypertension, dyslipidemia, osteoarthritis, and certain forms of cancer [3,4], making such chronic diseases responsible for approximately 39 million deaths annually world-wide [5].

Current evidence suggests that one of the most effective weight loss approaches is a change in dietary and physical activity behaviors through lifestyle modification programs (e.g., cognitive-behavioral treatment programs) [5-11]. Consequently, several lifestyle modification programs have been developed and implemented to promote healthy dietary changes and to increase physical activity [9-14]. The National Institutes of Health Diabetes Prevention Program (DPP) [10] has been found to be the most effective in producing health behavior changes and positive health outcomes. Findings from a DPP randomized controlled trial indicate that individuals lost on average 7% of their body weight through healthy eating and moderate physical activity (150 minutes/week) and reduced the incidence of diabetes by 58% [10]. It was more efficacious than pharmacotherapy (i.e., 31% reduction in diabetes using metformin) [10].

Sustaining the effects of the DPP lifestyle intervention program, however, has proven to be more challenging. After 2.5 years participants re-gained nearly half of the weight lost and their plasma glucose continuously increased [15]. The effectiveness of the program seems to decrease once the contact frequency with the lifestyle coaches decreases. Researchers suspect that participants may not have the tools at hand to sustain their behavior change for life (which is necessary to avoid regaining weight) without continued assistance and encouragement from a lifestyle coach [16]. Moreover, the DPP is costly because it consists of a large number of one-on-one sessions (16 weekly core sessions plus monthly follow-up sessions) delivered by highly trained professionals (at least master’s level) and strategies for motivating participants (e.g., paying for gym fees, exercise videos, etc.) when they have difficulty reaching their lifestyle goals. In response to these concerns, a modified and shortened version of the DPP has been developed by the same research group that developed the DPP: the Group Lifestyle Balance program (GLB) [17]. The GLB uses the same curriculum, and education and cognitive-behavioral techniques as the original program but has a less intensive delivery protocol (only one year, group-based, fewer sessions). It is therefore more cost effective to deliver [17,18]. A recent meta-analysis of all published GLB studies (*N* = 28) found an average weight loss of 4% body weight at 12 months [19]. While clinically important, this is less than the average weight loss achieved in the original DPP trial (7%) [10]. Given that every kilogram of weight lost was associated with a 16% reduction in risk for diabetes in the DPP trial [20], this difference in effectiveness is important. Further, one might also expect that participants regain weight at perhaps even larger rates than in the original DPP after program completion because the GLB is shorter and less intense. Thus, it is pertinent to increase the short- and long-term effectiveness of the GLB through means that compensate for the efficacy lost due to the group-based intervention delivery, lower contact intensity, and shorter duration.

In summary, the DPP is costly, which limits widespread dissemination. The group-delivered version (i.e., the GLB), on the other hand, is cheaper and can thus be disseminated more widely, but it is less effective. Weight regain occurs after both programs. For these reasons, we propose to enrich the GLB with techniques that will result in lasting behavior change. The techniques we propose to integrate into the GLB will facilitate the formation and maintenance of new habits. Ouellette and Wood [21] define habits as “[behavioral] tendencies to repeat responses given a stable supporting context” (p. 55). According to Ouellette and Wood, these cue-behavior chains have to be practiced in order to become automatic. Automaticity of the adopted behavior is the core aspect of habits [22]. Based on the literature of habit change, we have identified *if-then plans* and their *mental practice* as the two strategies for which strongest empirical evidence exists to suggest that they lead to lasting behavior change, i.e. the formation of habits. We have therefore integrated these habit formation techniques into the GLB. This paper describes the protocol of a randomized controlled trial that compares the enriched version of the GLB with its original version.

### If-then plans and mental practice

Two decades of research have provided strong evidence that if-then planning (implementation intentions) and mental practice are crucial techniques for creating strong and lasting habits [23-27]. If-then plans are concrete action plans that specify, in an if-then format, when, where, and how one will act in order to achieve a specific goal (“*If* situation Y occurs, *then* I will initiate goal-directed behavior X!” [26-29]). Forming if-then plans has been found to be much more effective than relying solely on motivation and willpower, as expressed in mere goal intentions (“I will do X”). In fact, a large meta-analysis reported medium-to-large effects of if-then plans on goal achievement across many behavior domains (94 studies, *d* = .65) [28,29]. In a subsequent meta-analysis reviewing physical activity studies, a medium average effect size difference was found [30]. Most importantly, longitudinal studies show that the formed habits are strong and more durable when using if-then planning [31-36]. In the study with the so far longest follow-up interval (48 months), it was found that adolescents in the if-then planning group showed a 34.5% reduction in smoking uptake [32].

Evidence suggests that if-then planning leads to lasting behavior change because the plans render behavioral responses automatic [37]. Specifically, automaticity is achieved because (1) specifying the exact cues in the ‘if’ component prompts the behavioral response that the person committed him/herself to when forming the if-then plan [37]; and (2) the if-then contingency format establishes a strong mental link of causality between the critical cues (‘if’ component) and the chosen behavioral response (‘then’ component) [38]. By explicitly making the causal connection between the critical cues and the goal-directed response through the if-then format, these plans effectively move people from planning to automatically carrying out the behaviours [38,39]. Once automatic, the behavioral response requires little mental effort, contemplative decision-making, self-regulation capacity, or external reinforcement to be carried out [40]. For instance, Milne et al. [36] found that of the people who made if-then plans about when, where, and how to exercise, 100% exercised at the place that they had specified in their if-then plan, 97% exercised at the time that they had specified, and 88% exercised on the day that they had specified.

Creating new habits for complex behaviors such as healthy eating or exercising requires frequent repetition of the same behavior over an extended period of time for it to become habitual. If-then plans can help people to repeat the same behavior over and over again and thereby form habits faster and more enduringly. Mental practice independently has also been shown to improve goal attainment (e.g., [41]). Mental practice (or mental rehearsal) is defined as ‘the cognitive rehearsal of a task in the absence of overt physical movement’ (p 481 [42]). To be effective, mental practice involves the use of multiple sensory modalities, such as imagining the motor movements, objects, situations, emotions through vision, smell, hearing etc. [43-45]. In our own research, we have shown that including mental practice into if-then planning significantly enhances the effectiveness of the if-then plans [23-25]. Combining if-then planning and mental practice is more effective than either if-then planning or mental practice alone [23]. For instance, average daily fruit consumption over a 7-day period more than doubled from pre- to post-intervention in a sample of undergraduate students (from 1.79 to 3.85 portions of fruit/day) after participants were trained to mentally practice their implementation intentions to eat more fruit. There was no increase in the control group [24].

Because of the strong evidence base for the effectiveness of if-then planning, mental practice, and their combination for goal attainment and habit change, we integrated these habit formation tools into the GLB to foster and accelerate the formation of healthy eating habits and increase regular exercise participation. Below, we describe the protocol of the randomized controlled trial that compares the enriched GLB with the standard GLB. The primary outcome will be the mean difference in percent body weight loss between the enriched and the standard GLB at 3, 12 and 24 months. The study is called the *McGill CHIP Healthy Weight Program*. It is conducted in collaboration with the McGill Comprehensive Health Improvement Program (CHIP), which is a multidisciplinary disease management and prevention program that is the primary site of academic research and teaching activities surrounding exercise and health for the McGill medical community. The specific study aims are:

1) To determine the effectiveness of the enriched GLB on weight loss at 3, 12, and 24 months (primary outcomes) and other weight relevant risk outcomes (secondary outcomes) at 3, 12, and 24 months following implementation compared with the standard GLB.

2) To determine whether habits are formed faster (3 months), are stronger at program completion (12 months), and are maintained for a longer period of time after program completion (24 months) in the enriched versus the standard GLB.

3) To determine at 3, 12, and 24 months following implementation whether the effectiveness of the enriched GLB is mediated by stronger habit formation.

## Methods/Design

### Trial design

The McGill CHIP Healthy Weight Program is a randomized controlled trial (RCT) with a parallel group design. Participants will be randomly assigned (1:1) to either the enriched or the standard GLB. Primary and secondary outcome measures are completed at baseline, after 3 months (completion of the 12 weekly sessions, core program), after completion of the program (12 months), and one year after completion of the program (24 months).

### Participants

Participants will be 154 men and women between the ages of 18 and 75 who are overweight or obese (BMI 28-45 kg/m^2^), exercising less than 200 min/week. The trial commenced with a lower BMI criterion of 27. This was changed 3 months into the trial to 28 in order to target a more clinically relevant population. Exclusion criteria include any limitation that would preclude full participation in the intervention or can have a confounding effect on the primary outcomes, specifically: (1) having been diagnosed with diabetes (hemoglobin A1c ≥7.0%); (2) taking the medication metformin (used for treating pre-diabetes or diabetes); (3) having been pregnant in the past 6 months or planning on becoming pregnant in the next 2 years; (4) currently undergoing treatment for cancer; (5) using medication that affects body weight (e.g., loop diuretics); (6) being unable to participate in regular moderate physical activity; (7) having severe uncontrolled hypertension (>190/100 mm Hg); (8) being unable to communicate in English or French; (9) being diagnosed with bulimia nervosa, currently active major depression, or other severe psychiatric disease (including dementia); (10) suffering from a heart attack, stroke, or heart failure within the past 6 months; (11) experiencing excessive weight loss (more than 10 pounds or 4.54 kilograms) in the past 3 months; (12) currently participating in another weight loss program; (13) having had bariatric surgery in the past 2 years or plans on getting it in the near future; (14) planning on moving away from Montreal within the next year; (15) having another member of one’s household enrolled in the program.

### Participant recruitment

Participants are recruited through: (1) flyers and information pamphlets that will be distributed at various clinics, hospitals, and pharmacies; (2) personal communications with the research team, including the nurses and physicians who refer patients to the CHIP and additional local physicians who are in contact with potential eligible participants; (3) emails to 700 individuals who previously were enrolled in one of CHIP’s programs; (4) emails to the members of the YW-YMHA in which the CHIP is located; (5) electronic newsletters of various universities and institutions around Montreal; and (6) advertisement through social media. Interested individuals will contact the research coordinator through email or the phone number published in the information materials.

### Determining eligibility

A 3-step approach for determining eligibility is used: In the first step, upon expressing interest in participation, potential participants receive an email containing more information about the study and a hyperlink directing them to a secure online screening questionnaire assessing the aforementioned inclusion and exclusion criteria. If respondents are not eligible, the online screening session is immediately terminated. Respondents are thanked for their time and participation and asked if they would like to be contacted by CHIP for other potentially suitable programs. In the second step, individuals who are identified as overweight and sedentary are then invited to have their hemoglobin A1c measured at CHIP and their BMI confirmed by examination. The blood sample drawn will be tested in a laboratory, and results will be available within 24 to 48 hours. If the individuals’ hemoglobin A1c level is below 7%, then they are eligible to participate in the study. In the third step, eligible participants participate in a medical exam and a graded exercise stress test (EST) to ensure that they can safely engage in moderate intensity exercise. At this appointment, baseline study measurements are also taken and participants are randomly assigned to one of the two intervention arms.

### Intervention

The intervention and data collection take place at the CHIP or on the downtown campus of McGill University. The GLB (in its standard or enriched form) will be delivered over one year (12 weekly core sessions, 4 transitional sessions over 3 months, and 6 monthly support sessions). Group sizes will comprise of approximately 6-10 individuals and the sessions will last for approximately one hour. Groups are led by trained lifestyle coaches. The lifestyle coaches are doctoral students in clinical health psychology and have completed the GLB 2-day training workshop at the University of Pittsburgh. The lifestyle coaches will have continuous support on as-needed basis from the Diabetes Prevention Support Center (DPSC) of the University of Pittsburgh.

The enriched GLB group will receive the exact same intervention with instructions for if-then planning and mental practice integrated into the intervention manual. Instructions for delivering if-then planning and mental practice are based on our previous studies [23-25]. Specifically, the concepts of if-then planning and mental practice are introduced to participants in Session 1 and practiced with the example of weighing oneself and tracking one’s food intake (see example in Additional file 1). Lifestyle coaches will guide participants through the formation of effective if-then plans using structured if-then plan handouts (see Additional file 2 for an example). The intervention manual and handouts are available from the authors.

### Compensation for participation and assessments

There will be no financial compensation for participating. Participants do not have to pay for the program. They will receive all program materials (pedometer, handouts, etc.) for free.

### Outcome measures

Measures assessed in the study are reported in Table 1 and described below. The *primary outcome for Aim 1* will be the mean difference in percent body weight loss between the enriched and the standard GLB at 3, 12, and 24 months. Weight loss was chosen as the primary outcome because it was the most important predictor of diabetes incidence in the large-scale diabetes prevention studies [9-11]. Diabetes incidence was reduced by 16% for every kg of weight loss in the original DPP [20]. Weight is assessed with a digital scale without shoes to the nearest 0.1 kg.

**Table 1:** Measures

**Outcomes**	**Variable**	**Measure/source**
**Primary outcome (Aim 1)**	**Mean difference in % body weight loss**	- Weight In Kg (Digital Scale)
**Secondary outcomes (Aim 1)**	**(1) Goal achievement**	
	a. % reaching weight loss goal	- Weight In Kg (Digital Scale)
	b. % reaching exercise goal	- Sum of min. over 7 days (tracking sheets)
	**(2) Diabetes risk factors**	
	a. Waist circumference	- Tape (cm)
	b. Hemoglobin A1c	- Immuno assay
	c. Blood pressure	- Mercury sphygmomanometer
	d. Total cholesterol/HDL ratio	- Enzymatic assay
	**(3) Physical activity**	
	a. Total duration	- Minutes (online tracking)
	b. Steps taken	- Pedometers (online tracking)
	c. Metabolic equivalents	- Ainsworth tables
	d. Aerobic fitness	- EST (test duration in minutes)
**Aims 2 and 3**	**Habit formation indices:**	
	**(1) Self-monitoring index**	
	a. Weight	- Days per week (0-7, online tracking)
	b. Fat grams	- Days per week (0-7, online tracking)
	c. Calories	- Days per week (0-7, online tracking)
	d. Physical activity	- Days per week (0-7, online tracking)
	**(2) Behavior change index**	
	a. Fat grams intake	- Daily average (online tracking)
	b. Calorie intake	- Daily average (online tracking)
	**(3) Habit strength index**	- Self-report index of habit strength
	a. Self-monitoring	
	b. Behaviors	
**Moderators**	**Socio-demographic variables**	- Self-report questionnaire
	Age, gender, ethnicity, education, socio-economic status, smoking status	

### Secondary outcome measures for Aim 1

Table 1 outlines the secondary outcome measures for Aim 1, which are: (1) Goal achievement of (a) the weight loss goal (7% of body weight) and (b) physical activity goal (150 min/week) at 3, 12, and 24 months; (2) measures of other health relevant outcomes measured at 3, 12, and 24 months, compared to baseline levels; and (3) physical activity and aerobic fitness assessed using 4 methods to capture different aspects of physical activity and aerobic fitness, namely: (a) how many minutes of physical activity participants engaged in during the day (online tracking), (b) the number of steps taken during the day recorded using a pedometer, and (c) the metabolic equivalents (MET) of the different physical activities over 7 days translated into step equivalents using the tables developed by Ainsworth [46]. Participants will track their weight changes and physical activities online through CHIP’s myhealthcheckup.ca website, and their food choices through the website of the Dieticians of Canada, eaTracker.ca, through the MyFitnessPal application, or on paper.

### Measures for Aims 2 and 3

Aim 2 of the study is to determine whether habits are formed faster (3 months), are stronger at program completion (12 months), and are maintained for a longer period of time after program completion (24 months) in the enriched as compared to the standard GLB. Aim 3 of the study is to determine at 3, 12, and 24 months following implementation whether the effectiveness of the enriched GLB is mediated by stronger habit formation. Habit formation will be assessed using the following indicators (see Table 1): (1) Adherence to self-monitoring in relation to: (a) weighing themselves, (b) fat grams consumed, (c) calories consumed, and (d) physical activity; (2) Degree to which behaviour change occurred will be measured by the (a) fat gram intake (as measured by the mean self-reported daily intake recorded through online or paper tracking over the course of the assessment week), and (b) calorie intake (as measured by the mean self-reported daily intake recorded through online or paper tracking); (3) Degree of habit strength will be assessed for (a) self-monitoring and (b) carrying out behaviors with the Self-Report Index of Habit Strength (SRHI) [47], with a focus on automaticity, which reflects the active ingredient of habits [47]. The SRHI is a 12-item measure that assesses empirically derived features of habits (e.g. degree of repetition, automaticity) on rating scales ranging from 1 (*disagree*) to 7 (*agree*). The SRHI has been shown to have high internal consistency (alpha = .89), test-retest reliability (*r* = .91), and divergent and convergent validity [22]. The scale was adapted for the purposes of this study and is available from the authors.

### Sample size

The primary outcomes are the differences in mean percentage weight loss between the enriched and standard GLB at 3, 12, and 24 months. The recent systematic review and meta-analysis by Ali et al. [19] of all GLB studies conducted so far allowed for calculating the mean % weight loss achieved in studies with follow-ups at 3 months (*N* = 11 studies) and at 12 months (*N* = 16 studies). Results showed a mean percent weight loss from baseline of 4.37% at 3 months and 4.35% at 12 months. Meta-analysis data for longer time frames are not available. The Finnish Diabetes Prevention Study, however, found a weight regain of around 33% at 2 years after completion of the intervention. Therefore, in the standard GLB, the expected percentage weight loss at 24 months (from baseline) is 2.92%. Based on the effect sizes in our preliminary data [23-25,34], we expect an additional weight loss of 3% at all three time points when if-then plans and mental practice are added to the program (possibly more at 24 months because we are expecting less weight regain). Based on a meta-analysis of past GLB studies [19], we expect the standard deviations for percent weight loss to be 4.3 in the enriched and standard GLB groups at the 3 time points. Using these inputs, a sample size of 64 participants per group is sufficient to estimate the between-group difference to an accuracy of ± 1.5% and a 95% CI at all 3 time points. To account for a potential drop out of around 20% (in line with comparable studies), we are increasing the sample size to *N* = 154, *n* = 77 per group.

### Randomization

To randomize participants to the two intervention arms, a randomization sheet generated by a random digit generator is used (http://www.randomizer.org). Throughout the recruitment process, the list of randomized numbers will be assigned to participants by the research coordinator in sequential order from 1 to 154 in the order in which participants completes the baseline CHIP appointment. Once a participant is assigned a condition, the research coordinator enters the date and the participant’s initials next to the condition on the randomization sheet. This information together makes up the participant’s anonymous intervention code. Participants are told that the study evaluates two different approaches to lifestyle changes to see if one is superior to the other. Throughout the recruitment process, groups of approximately 6 to 10 people will be created according to their language (i.e., French or English), location, and time of day preferences. It was inherently not possible to blind the interventionist to interventions. However, the staff assessing the outcome variables (e.g. weight, EST) is blind to which intervention the participants were assigned.

## Trial status

The RCT began participant recruitment in April 2013 and the first groups started in November 2013. Recruitment is currently (May 2014) ongoing until the required sample size is reached.

## Discussion

In order to meet the large needs for weight loss programs, effective group-delivered, shortened versions are required. The described intervention protocol provides a detailed description of the content and delivery of an RCT aimed to test the effectiveness of an enriched GLB program. Incorporating habit formation techniques could ultimately allow reducing the contact frequency and length of lifestyle modification programs, which would reduce their costs and enable wider dissemination. The habit formation tools integrated into the standard GLB are implementation intentions and mental practice, techniques proven effective for changing and sustaining new health behaviors.

## Competing interests

The authors declare that they have no competing interests.

## Authors’ contributions

BK, IL, LJ, AL, and SG conceived the study. All authors participated in its design and coordination. BK, EI, ZX, and MC helped to draft the manuscript. All authors read and approved the final manuscript.

## Pre-publication history

The pre-publication history for this paper can be accessed here:

http://www.biomedcentral.com/1471-2458/14/470/prepub

## Supplementary Material

Additional file 1 Keeping track. Click here for file

Additional file 2 If-then plan for weighing yourself. Click here for file
