# Supplementary material for: Increasing the effectiveness of the Diabetes Prevention Program through if-then plans: study protocol for the randomized controlled trial of the McGill CHIP Healthy Weight Program
Source: BMC Public Health. 2014 May 18;14:470. doi: 10.1186/1471-2458-14-470 (PMC4032631; doi:10.1186/1471-2458-14-470)
Supplement: Additional file 1 — Keeping track. [file 1471-2458-14-470-S1.docx]

**Additional file 1**

**Keeping Track**

You may initially find it difficult to remember to weigh yourself each week or day and to record your weight. Don’t worry, this is a common experience and we will work on this step by step until it becomes a solid habit. Psychologists have found certain tools that can help you form a new habit: They have found that it helps tremendously to make a very concrete action plan, that is, to specify in detail **when, where, and how** you will do it - we will also refer to them as **if-then plans**. For example, stating that “When I take a shower on Monday morning, then I will weigh myself right after” will remind you to do it every Monday morning and not to forget about it.

We will make a number of if-then plans throughout this program and **they are the single most important tool that you will be using to lose weight and exercise more**. Numerous studies have shown that if-then plans are very effective. For example, one study found that participants in a weight loss program who made if-then plans about their eating habits lost twice as much weight as participants who did not make if-then plans. Another study found that when people made if-then plans about when, where, and how they would exercise, 100% of them exercised at the place that they had decided in their if-then plan, 97% exercised at the time that they had planned to do it and 88% exercised on the day they had specified in their plan.

With the help of if-then plans, the weekly or daily weighing will become routine and automatic so that you will no longer even have to think about it. If-then plans are one of the most valuable tools that we will be using throughout all the sessions for behaviours you want to change.

Let’s practice how you can use if-then plans to achieve your goal for weighing yourself for next week. Try to think of a routine for weighing yourself, in other words, always doing it at the same time and in the same situation. An example would be: “When I come into the bathroom on Monday morning after waking up, then I will weigh myself.” Now please go to “if then plan” section of our notebook, and follow the steps of making an if-then plan for weighing yourself with me.

[As you explain the steps by giving the following examples, allow a bit of time for each step for participants to enter the information.]

1. **Choose a habit you want to establish**

- Let’s take the example of weighing yourself every week or every day

1. **Establish a new routine** *(When, where, and how will you do it?)*

- *When* is the best time during the day for you to weigh yourself?
- I recommend that you weigh yourself on a specific day (Monday would be good) and almost at the same time (e.g. after taking a shower in the morning)
- *Where* - help participants generate not only the time but also where they would do it.

1. **Minimize barriers** *(What could hinder you from doing it and how can you overcome it?)*

- You may wake up late and feel that you don’t have enough time to weigh yourself before you go out to work. But remember that weighing wouldn’t take more than a few seconds. [If they come up with too many disruption, brainstorm to generate another time/routine.]

1. **Form if-then plan for weighing yourself**

You may want to make statements like:

- - 1. When I come into the bathroom on Monday morning, then I will weigh myself and record it.

**Form if-then plan for dealing with the barrier**

- - 1. If I feel that I don’t have time to weigh myself, then I will remind myself that it will take only a few seconds.

So, that was an example of an if-then plan. We will develop a few of these together throughout our sessions.

1. **Mentally practice the plan:**

To make it easier for you to change your habits, I want to share another tool with you. This tool is **mental practice of your if-then plan**, that is, to mentally imagine in great detail how you will carry out your if-then plan. Imagining how it will look like, sound like, feel like, and smell like will increase your chances of achieving your if-then plan.

Research from the field of neuroscience suggest that imagining an action is the same as actually carrying it out because it lights up the same areas in the brain. In fact, mental practice is a very popular method used in sports psychology. Before an athlete takes on a new and difficult movement, they imagine it first, rehearsing all the steps mentally before trying it for the first time.

Now look back at the if-then plan you have just formed for weighing yourself; imagine **when** (e.g. time of day) and **where** (e.g. bathroom, bedroom) you will do it (e.g. weigh yourself and record your weight in the Daily Weight Log sheet after waking up in the morning). Imagine the details of the objects involved in your plan, for example the scale – its shape, size, and color – and what you would do (e.g. stepping onto the scale before taking a shower in the morning). Really try to take a moment to close your eyes and imagine the if-then plan for weighing yourself in this very detailed and vivid manner. Make sure to use all of your senses! Perhaps imagine yourself yawning while stepping on the scale because you just got up…!

Given that we made our first if-then plan and that you have mentally imagined the plan as a group, if at any point in your mental practice you find something doesn’t make sense to you (e.g., you shower in the evenings instead of in the mornings) you can make adjustments to your personal if-then plan so that it makes more sense to you (e.g., weighing yourself after you brush your teeth).

Mental practice is also very useful when it comes to imagining **your feelings after you actually did what you want to do** or after you achieved your goal. For example, try to imagine how happy you will be when your scale is showing you a smaller number each time you measure yourself, or finally the weight that you want to achieve as part of your weight loss plan, or that you are taking charge of health and weight. Even though you may not see drastic changes in your weight each time you step on the scale, imagining the positive outcomes (e.g., feeling and having more energy, playing with your kids or grandkids, etc.) will keep you motivated to work toward your goals and remind you of what is possible if you maintain your effort.

[Ask the group to close their eyes and to vividly imagine every aspect of the plan that they created above. Make sure to do the following:

Ask the group to close their eyes and to vividly imagine every aspect of the plan that they created above. Make sure you ask participants to use all their senses, etc. The purpose of this session is to have all the participants practice mental imagery.

Ask participants how they found the mental imagery, were they able to image, did they have to make changes to their if-then plan?]

Once you have mentally rehearsed your plan, you can rate how confident you feel about your plan. Only plans that you feel the most confident about achieving will be transferred to the if-then plan summary sheet.

**6. Assess your confidence** *(For each if-then plan, rate how confident you are that you will be able to carry out the plan using a number from 1 to 7, where 7 means “very confident”.)*

**Confidence if-then plan: _____**

**Confidence barrier plan: _____**

Revise your plans if you gave it a rating of 4 or lower and make them easier to do.

**7.** **Transfer the plans to the if-then summary sheet.**
