# Supplementary material for: Increasing the effectiveness of the Diabetes Prevention Program through if-then plans: study protocol for the randomized controlled trial of the McGill CHIP Healthy Weight Program
Source: BMC Public Health. 2014 May 18;14:470. doi: 10.1186/1471-2458-14-470 (PMC4032631; doi:10.1186/1471-2458-14-470)
Supplement: Additional file 2 — If-then plan for weighing yourself. [file 1471-2458-14-470-S2.docx]

**Additional file 2**
 **If-Then Plan for Weighing Yourself**
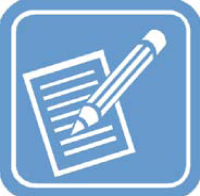


**If-then plans** specify when, where, and how you will do something. Throughout the program, you will be building a number of if-then plans every session, and practice them daily. This will ensure that the eating and exercise changes you will make will be lasting, i.e. become your new lifestyle habits.

**Build your own if-then plan for weighing yourself:**

1. **Which new habit do you want to establish?**

Weighing myself regularly and often.

1. **When, where, and how will you do it?**

_________________________________________________________________________

1. **What could hinder you from doing it (could be a barrier) and how can you overcome it?**
   _________________________________________________________________________

Solution:_________________________________________________________________

1. **Form if-then plan for weighing yourself:**

*If/when__________________________________________________________________,*

*then I will weigh myself.*

**Form if-then plan for dealing with the barrier:**

*If/when__________________________________________________________________,*

*then_____________________________________________________________________.*

1. **Mentally practice the plan and modify it if necessary.**
2. **Assess your confidence** *(For each if-then plan, rate how confident you are that you will be able to carry out the plan using a number from 1 to 7, where 7 means “very confident”.)*

**Confidence If-Then Plan: _____**

**Confidence Barrier Plan: _____**

Revise your plans if you gave it a rating of 4 or lower and make them easier to do.

1. **Transfer the plans with ratings of 5 or higher to the if-then summary sheet.**
